# Inequality, Education, Workforce Preparedness, and Complex Problem Solving

**DOI:** 10.3390/jintelligence6030033

**Published:** 2018-07-16

**Authors:** Patrick C. Kyllonen

**Affiliations:** Educational Testing Service, Princeton, NJ 08648, USA; pkyllonen@ets.org; Tel.: +1-609-734-1056

**Keywords:** inequality, problem solving, complex problem solving, PISA, National Assessment of Educational Progress, collaborative problem solving, skills, general ability, cognitive ability, general fluid ability

## Abstract

Economic inequality has been described as the defining challenge of our time, responsible for a host of potential negative societal and individual outcomes including reduced opportunity, decreased health and life expectancy, and the destabilization of democracy. Education has been proposed as the “great equalizer” that has and can continue to play a role in reducing inequality. One means by which education does so is through the development of complex problem solving skills in students, skills used to solve novel, ill-defined problems in complex, real-world settings. These are highly valued in the workforce and will likely continue to be so in the future workforce. Their importance is evident in results from employer surveys, as well as by their inclusion in large scale international and domestic comparative assessments. In this paper, I review various definitions of complex problem solving and approaches for measuring it, along with findings from PISA 2003, 2012, and 2015. I also discuss prospects for teaching, assessing, and reporting on it, and discuss the emerging importance of collaborative problem solving. Developing and monitoring complex problem solving skills, broadly defined, is a critical challenge in preparing students for the future workforce, and in overcoming the negative effects of inequality and the diminishment of individual opportunity.

## 1. Introduction

In 2013, former U.S. president Barack Obama argued that reducing economic inequality and improving upward mobility was “the defining challenge of our time” [1]. This is not a uniquely American perspective. Similar sentiments have been expressed by Prime Minister Justin Trudeau of Canada [2] and the topic has been central in recent elections in Germany, Italy, and France. Best sellers by the economists Thomas Piketty [3] and Joseph Stiglitz [4] chronicle the growing concentration of income and wealth and its implications for reducing opportunity, decreasing health and life expectancy, and destabilizing democracy. 

Since Horace Mann [5], education has been seen as the “great equalizer,” a sentiment later echoed by Obama’s Secretary of Education, Arne Duncan [6]. For example, log wages go up linearly with years of education, from 7th grade to Ph.D. [7]; estimates of the causal effects of education on earnings suggest roughly 10% per year of education [8] with even higher returns to those from disadvantaged backgrounds [7])^1^. Thus, among the remedies to the growing inequality problem proposed by Obama was increasing educational attainment. He proposed doing this through greater spending on high-quality pre-school, emphasis on career and technical education, and making higher education more affordable. Education not only affects earnings, but brings a host of other benefits, including increased life satisfaction, trust, and social interaction, and improved decision making about choices related to health, marriage, and parenting [9].

It is important to consider what it is about education that serves to deliver these positive benefits. A way to think about this is that education serves two functions that are extremely valuable in the workplace and in life generally. Education teaches domain-specific skills, particularly mathematics and language skills, but also more specialized curricular skills such as those associated with particular occupations (in career and technical education) or college majors. In addition, education teaches domain-general skills such as problem-solving, communication skills, and conscientiousness.^2^ Certainly mathematics and language skills are essential core skills for continued education and they are the focus of many large-scale surveys designed to report on the quality of nations’ education systems (e.g., NAEP and PISA), and workforces (PIAAC). However, a theme here is that an emphasis on domain-specific skills may be disproportionate, at the expense of the domain-general skills. Consider that there are many occupations at various levels that require minimal mathematics skills (e.g., court reporters, law clerks, radio announcers, and historians) or minimal language skills (janitors, sewing machine operators, and watch repairers). Further, many people are employed in jobs outside their academic major—Robts [12] found that 45% of college graduates reported that their job was only partially related or not related to their field of study. He also found that the costs of working in an out-of-field job were lowest for liberal arts majors, that is, those probably most likely to learn domain-general skills.

Another issue pertaining to the relative importance of domain-specific vs. general skills is that the workforce keeps changing, primarily due to technology, and the pace of change appears to be accelerating. For example, technologies such as the telephone and electricity took four to six decades from invention to 40% market penetration, whereas tablets and smartphones accomplished this within a decade [13]. This has implications for the nature of work in the future workforce. A National Research Council workshop and report [14] on the future labor force highlighted the importance of broad general skills (social, interpersonal, and problem solving) as initially defined in the Department of Labor’s Secretary’s Commission on Achieving Necessary Skills (SCANS) report [15]. Their importance was not limited to college, but included Career and Technical Education (CTE) as well. Ken Kay, a speaker at the workshop said that today’s students will need broad skills (communication, creativity, problem solving), to prepare for “multiple careers and multiple jobs” [14] (p. 88), and as a “self-defense mechanism” in case of displacement or layoffs due to disruptions in the workforce.

In sum, education is the “great equalizer” that can mitigate the deleterious effects of inequality and the concentration of wealth on society and on individual opportunity. A proposal is that a mechanism by which education does so is through the development of general, complex problem solving skills, which may at least partly be a byproduct of instruction in science, technology, engineering, and mathematics, which occurs in K-12 [16], community college [17], higher education [18,19], and in career and technical education [20]. In this commentary, I explore definitions of problem solving and complex problem solving, review its importance, discuss its malleability, and conclude with suggestions on how it might be monitored and developed in school so as to enhance the great equalizing effects of school.

## 2. Complex Problem Solving

Complex problem solving (CPS) skills may be defined broadly as those “used to solve novel, ill-defined problems in complex, real-world settings” [21].^3^ Complex problem solving describes the activities associated with some of the most demanding and highly financially valued and rewarded occupations in the workforce, such as chief executives, emergency management directors, judges, physicists, surgeons, biomedical engineers, and neurologists (based on data from the U.S. Department of Labor’s Occupational Information Network, O*NET [21]). There is every indication that complex problem solving skills are likely to continue to be valued in the future workforce [22]. Students with strong complex problem solving skills are in demand by employers for entry level positions [23,24] and jobs requiring complex problem solving are the ones that pay the most throughout one’s career [21].

However, before considering issues of how to develop and evaluate complex problem solving (CPS) skills in students, there is a question about what exactly complex problem solving skill is. Can CPS even be considered a general skill, or is it simply a name for a broad set of specialized skills in diverse knowledge domains. Both the chief executive and surgeon draw on CPS skills, according to evaluations by job experts, but what do skills in the two domains have in common, if anything? If one acquires complex problem solving skills in one domain, through school or elsewhere, to what extent do such skills transfer to different knowledge domains? While complex problem solving skills are most important in occupations such as chief executives, physicists, and chemical engineers (for these jobs “complex problem solving” has a rated importance level of 78 to 85 on a 0–100 scale [21]), complex problem solving is also rated as important, just not as important, in a broad variety of other occupations such as art directors, fashion designers, biologists, materials engineers, and survey researchers (importance level 66 on a 0–100 scale), and even in so called middle-skills jobs such as medical assistants, license clerks, crane operators, and retail salespersons (importance level 47 on a 0–100 scale). What is common about complex problem solving skills across both domains and levels of occupations? Is “CPS skills” even a coherent psychological construct or is it simply a shorthand label, similar to “expertise,” for unrelated activities which have only in common that they are difficult?

The answers to these questions have implications for how complex problem solving can be taught and developed, and whether CPS skills should be targeted for instruction generally, or in the context of specific knowledge or curricular domains. If complex problem solving skill is a general skill, then perhaps it makes sense to teach it directly, using examples from different knowledge domains. If complex problem solving skill is simply a label for a diverse set of activities that have nothing in common, other than a label, that suggests that there will be little transfer of skill, neither from school to the workplace, nor from one job domain or subject matter domain to another.

The issue of the domain specificity of what appears to be a very general skill is a hotly debated topic, not yet settled, with advocacy on both sides of the issue. In this article, I address the issue of the nature of complex problem solving skill, and the issue of the degree to which it may be developed in school, in training, or on the job. I begin with a summary of the indications that it is a highly valued skill, based primarily on surveys and other informal reports.

## 3. Employers Seek Complex Problem-Solving Skills Now and Likely in the Future

Several surveys have been conducted, which ask employers what skills they look for in recent graduates during hiring or what employers’ greatest needs are. The National Association of Colleges and Employers annually surveys its U.S. employer members on a variety of topics to project the market for new college graduates. Table 1 presents the 2017 findings. “Problem solving skills” is the second most important skill category (behind “ability to work in a team”). Findings on the skills employers are looking for do not vary much from year to year, regardless of wording. For example, on the 2015 survey, the two highest “candidate skills/qualities” were “ability to work in a team structure” and “ability to make decisions to solve problems,” both of which received average ratings of 4.61 on a 1 (“not at all important”) to 5 (“extremely important”) scale [23] A similar survey [25], Table 2; 431 employers identified “critical thinking/problem solving” as among the top five “very important” applied skills for job success for new workforce entrants at all education levels (High school, two-year and four-year graduates).

These results are similar to those obtained by the Department of Labor and the states. For example, North Carolina’s Association of Workforce Development Boards [26] conducted a survey of employers (1152 respondents) that identified the greatest need to be soft skills, particularly communication/interpersonal skills (59%), critical and analytical thinking (47%), and problem solving (45%). Table 1 shows that a similar list of skills was obtained in a much larger study by McKinsey & Company, which surveyed employers from nine socioeconomically and culturally diverse countries (including the U.S.). Here, again, “problem solving” was rated as among the most important skills employers look for in recent hires. 

The U.S. Department of Labor’s O*NET program surveys employers, job incumbents, and occupational experts and analysts continuously^4^ on the abilities, interests, values, work styles, skills, tasks, education requirements, and other factors associated with 974 occupations covering the entire U.S. economy [27]. Among the over 200 ratings completed on each occupation are ones on the importance and level required of “complex problem solving”^5^ one of several cross-functional skills.^6^ Across all jobs, CPS is one of 14 skills (of 35) considered relevant to all occupations [28] (Table 1). The importance of CPS for an occupation is highly correlated with earnings for that occupation *r* = 0.42 with log median wages; [29], comparable to the relationship with general ability/general fluid ability (*r* = 0.39). Burrus et al. [30] identified a problem solving factor (primarily defined by complex problem solving and judgment and decision making ratings) from a factor analysis of all of the O*NET skills and abilities ratings. The problem solving factor had the second highest correlation with wages (*r* = 0.58) behind a communication skills factor (*r* = 0.60), but ahead of achievement/innovation (*r* = 0.46) and fluid intelligence (*r* = 0.41).

The preceding analysis suggests that complex problem solving skill is considered a very important skill in the workforce and among the most highly compensated skills. What about the future workforce? Autor, Levy, and Murnane [22] showed that, since the advent of computers and widespread automation, circa 1980, some jobs have grown and some declined, explained by computers substituting for human workers in performing routine cognitive and manual tasks (ones accomplished by following explicit rules), but complementing workers in “carrying out problem-solving and complex communication activities (‘non-routine’ tasks)” [22] (p. 128), a phenomenon known as job polarization. Subsequent research has reinforced those findings with other data sources, and emphasized the mutual importance of both [31,32]. As technology improves, it increasingly replaces work that can be automated. What remains are tasks that are difficult to automate, those requiring “flexibility, judgment, and common sense” [33], or “generalist occupations requiring knowledge of human heuristics, and specialist occupations involving the development of novel ideas and artifacts” with examples being chief executives, lawyers, and engineering and science occupations [34] (p. 40).^7^

If we adopt the definition of complex problem solving as skills used to solve novel, ill-defined problems in complex, real-world settings, then it would seem that these are indeed the skills most resistant to automation, and therefore likely to continue to be rewarded in the workplace. It is difficult to predict technology developments and their impact on the future workforce.^8^ However, there does seem to be some consensus around the idea that complex problem solving, broadly defined, and particularly when paired with communication skills, is likely to continue to be a valued skill, a conclusion in line with recommendations going as far back as the 1991 report of the Secretary of Labor’s Commission on Achieving Necessary Skills (SCANS [13]), though to a National Research Council workshop on future skill demands [12], and up to the current views reviewed here.

## 4. What Is Complex Problem Solving?

To this point, the terms problem solving and complex problem solving have been treated almost interchangeably. This partly reflects common usage in employer surveys and the economics literature, which typically do not make a distinction between them. However, there is a distinction in their usage within the psychological literature. It is useful to describe both terms and highlight the distinctions. 

### 4.1. Traditional Problem Solving

Traditional problem solving has a long history in psychology. Problem solving tasks include classic insight problems, ones characterized by an “aha” experience when realizing the correct answer [38], often found in riddles and puzzles books. Examples include retrieving out-of-reach bananas [39] (a study of monkeys), connecting nine dots with four lines [40], and attaching a burning candle to a wall with a box [41]. Problem solving tasks also include non-insight problems, or analytic problems, which are characterized as having a search space (with a starting state, goal state, and operators), such as Tower of Hanoi, Rubik’s cube, Chess, and missionaries and cannibals. They also include optimization problems such as the traveling salesman problem; inductive reasoning problems such as rule induction; and deductive reasoning problems such as verbal arithmetic (also, cryptarithmetic, [42]).^9^ There is some evidence for a lack of a distinction between insight and analytic problems, as Raven’s Progressive Matrices scores have been found to predict solution on the two types equally well [43]. 

Studies of problem solving have identified phenomena that impede or facilitate solution such as functional fixedness, mental set, and the importance of problem representation, and problem-solving strategies such as means-ends analysis, breadth vs. depth first search, working backwards, divide-and-conquer, trial-and-error, and reasoning by analogy [44]. A focal point of traditional problem solving research has been on teaching problem solving by making students aware of these kinds of phenomena and problem solving methods, which began with George Polya [45]. He focused on mathematical problem solving, and proposed that it follows the steps: (a) understand the problem; (b) devise a plan; (c) carry out the plan; and (d) review and extend the method used. Bransford and Stein [46] proposed a similar model, IDEAL (Identify, Define, Explore, Act, Look), designed to help schools and organizations teach problem solving.

These methods, distinctions, and problem solving strategies have served as the basis for frameworks and test specifications for OECD’s PISA problem solving assessment in several cycles, PISA 2003 [47], 2012 [48] and 2015 [49]. It is instructive to study PISA’s definition and implementation of problem solving assessments because: (a) the definitions are constructed by problem solving expert groups (which tend to change membership from cycle to cycle to some degree) representing international scientific consensus; and (b) the definitions and implementations are agreed to by the participating nations, OECD and non-OECD. Thus, PISA represents a fairly broad and common understanding, both scientifically and from a policy making perspective of what problem solving is. 

In PISA 2003, problem solving was defined as follows:

“Problem solving is an individual’s capacity to use cognitive processes to confront and resolve real, cross-disciplinary situations where the solution path is not immediately obvious and where the literacy domains or curricula areas that might be applicable are not within a single domain of mathematics, science or reading” [47] (p. 154). 

The definition highlights several features of problem solving as it is tested in PISA, both in the 2003 cycle and beyond, and as it is probably largely understood outside of PISA, by policy makers and stake holders in education and in the workforce communities internationally. The definition highlights: (a) “real situations” as opposed to more abstract formulations of problem solving, reflecting PISA’s position as a literacy examination emphasizing transferable skills; (b) the “non-obvious” nature of solutions, reflecting a general requirement for a task to be considered problem solving, and reflecting the non-routine nature of problem solving; and (c) the cross-curricular or domain-independent focus of the assessment, which draws on diverse (though not inaccessible) knowledge for problem solution. 

In PISA 2003, the problem solving framework was organized by problem types (decision making, system analysis and design, and troubleshooting), contexts (personal life, work and leisure, community and society), disciplines (math, science, literature, social studies, technology), processes (*understanding*, characterizing, *representing*, *solving*, *reflecting*, and communicating) (steps overlapping Polya’s [45] are italicized), and reasoning skills (analytic, quantitative, analogical, and combinatorial reasoning). The actual test included multiple choice, closed constructed response, and open constructed response item types, enabling both right-wrong and partial-credit scoring. 

PISA 2012 adopted a definition similar to that from PISA 2003 (see Appendix A, which lists problem solving definitions through several PISA cycles as well as the PIAAC definition), and the framework was largely the same. However, there were two major changes. One was that computer administration, enabling human–computer interactions, was implemented. The other was that a certain kind of interactive task, known as MicroDYN [50] and MicroFin [51], was introduced to constitute about two thirds of the assessment items. These item types involve manipulating variables to determine effects on outcomes, for example, manipulating temperature and water to observe effects on plant growth (these are example tasks from the U.S. National Assessment of Educational Progress, Interactive Computer Tasks (Science) assessment [52]). Despite these changes in the assessment, problem solving again correlated highly with the Mathematics, Reading, and Science assessments, the correlations being *r* = 0.81, 0.75, and 0.78, respectively [53]. Despite the high correlations, there were differences. The countries doing better in problem solving than expected (based on their Math, Reading, and Science scores, and expressed in standard deviations) were Korea (+0.12 points), Japan (+0.11), Serbia (+0.11), the U.S. (+0.10), and Italy (+0.10). Those doing worse than expected were Bulgaria (−0.54), Shanghai (China) (−0.51), Poland (−0.42), UAE (−0.42), and Hungary (−0.33).

PISA 2015 also largely adopted the problem solving approach and framework from the earlier assessments, but introduced a collaborative component. The major problem solving stages were retained (Exploring and understanding; Representing and formulating; Planning and executing; and Monitoring and reflecting), but now crossed with collaborative competencies (Establishing and maintaining shared understanding; Taking appropriate action to solve the problem; and Establishing and maintaining group organization). The collaboration was not truly authentic: The collaborator was a computer agent, and the test taker communicated with the agent by selecting multiple-choice responses in a chat window on the display screen, but it was collaboration nevertheless. Collaborative problem solving scores were, similar to previous assessments, highly correlated with Math, Reading, and Science scores, *r* = 0.70, 0.74, and 0.77, respectively (note that the correlations with Reading and Science were almost identical to the correlations found with the non-collaborative problem solving of PISA 2012, but with math, lower). In addition, the countries that did better and worse than expected were similar: Japan (+0.23), Australia (+0.23), U.S. (+0.22), New Zealand (+0.21), and Singapore (+0.17), vs. Russia (−0.22), Turkey (−0.19), Montenegro (−0.18), Tunisia (−0.17), and China (−0.17) [49] (p. 80). An intriguing finding was that the collaborative version of problem solving (in PISA 2015) differed dramatically from the non-collaborative (PISA 2012) version in one respect: in non-collaborative problem solving, boys outperformed girls in most countries; in collaborative problem solving, girls outperformed boys in every country by an average effect size of 0.3 [49] (p. 95).

PISA provides the common, consensus understanding of problem solving, and to some extent how it can be assessed in individuals. There is an alternative approach to understanding problem solving, and that is to observe how it is treated in the human abilities literature [54]. Specifically, to what extent do measures of problem solving correlate with other ability measures and how is that represented in factor analyses of abilities? Many tasks that might be considered traditional problem solving tasks, such as rule induction, were in fact included in Carroll’s [54] comprehensive analyses of abilities tests, and from those analyses problem solving would appear to be close conceptually to fluid ability, or general fluid ability (Gf) [29]. In addition, the problem solving tests used in PISA are typically highly correlated with the other tests of mathematics, reading, and science. As such, this indicates that problem solving is a fairly general factor, as would be expected to the degree that it aligned with general fluid ability. For example, PISA 2003 [47] (p. 189) problem solving scores correlated *r* = 0.89, 0.82, and 0.78 with mathematics, reading, and science scores, respectively (latent correlations), which places problem solving at the center of the four domains in the sense of having the highest average correlations with the other three domains. This, again, is consistent with the idea that problem solving and general fluid ability are closely related [29].

### 4.2. Complex Problem Solving

There are at least three definitions of complex problem solving. One is that it is the same as problem solving, but perhaps emphasizing task complexity or item difficulty, in the way that complex mathematics problems are difficult ones (see [55] for a discussion of the difficulty-complexity distinction). Arguably, PISA treats the concepts of problem solving and complex problem solving in this way. A second definition is implied in O*NET’s use of the term complex problem solving, quoted at the beginning of this article, which emphasizes “complex, real-world settings” as would be found in a job, such as CEO or surgeon. This is appropriate for O*NET, in which analysts rate the degree to which a job requires complex problem solving. The complex problem solving rating scale provides rating anchors: at the low end are jobs that require one to “layout tools to complete a job,” at the middle level to “redesign a floor layout to take advantage of new manufacturing techniques,” and at the high end to “develop and implement a plan to provide emergency relief for a major metropolitan area.”

A third definition of complex problem solving is a particular one that has emerged as a school of research, primarily in Germany [56,57] and it is useful to explore this definition in more detail. Dörner and Funke [58] characterize the distinction between regular and complex problem solving as one between well-defined and ill-defined problems, with well-defined ones having “a clear set of means for reaching a precisely described goal state” and ill-defined ones having “no clear problem definition, their goal state is not defined clearly, and the means of moving towards the (diffusely described) goal state are not clear.” Investigations of complex problem solving under this definition primarily involve computer simulated microworlds (as in the PISA 2012 MicroDYN and 2009 NAEP Science interactive computer task problems described above). Funke [59] argued that for problem solving to be considered truly complex, it should: (a) involve many variables; (b) that have mutual dependency; (c) be dynamic (changing over time); (d) at least partly intransparent (not possible to know the value of all variables at any one time); and (e) polytelomous, that is that there can be multiple and even conflicting goals for the problem solver. Newer definitions add even more features, such as: (f) involving self-regulation; (g) creativity; (h) combining solutions; and (i) in a high-stakes setting [58]. There is an issue of how this “German school” definition of complex problem solving aligns with a consensus definition (as illustrated in the PISA discussion, and as used in O*NET surveys). It may in some sense simultaneously be too narrow (not all complex problem solving has these features) and too wide (particularly with the latter feature additions, it may describe complex cognition rather than complex problem solving), but the exercise is informative and has led to considerable research activities exploring various notions of complex problem solving.

There are microworlds built based on the “German school” definition, particularly aspects “a” through “e,” but such microworlds tend to take a long time for students to learn and to get useful performance measures from. For example, microworlds from “the early years” were systems with between 20 and 2000 variables [58]. Consequently, shortened versions of microworlds that sacrifice some of these features (e.g., MicroDYN) have taken their place in PISA and other research contexts. A pertinent question asked by Dörner and Funke [58] is “what can we learn about BIG P by studying little p?” meaning what can we learn about complex problem solving in the complex, ill-defined, sense (BIG P) by studying shortened, simplified problem solving tasks (little p)?

## 5. Potential Uses of Complex Problem Solving Assessments

It is useful to review potential uses of a complex problem solving assessment. One is to monitor student growth and development, as PISA and PIAAC do for nations and systems over time, and as NAEP, PISA or a NAEP or PISA-like assessment could do in a longitudinal study to track student skills growth over grades. Another is to use complex problem solving assessments as selection instruments for college admissions, college placement, scholarships, military selection and classification, and employment screening. A third is to use complex problem solving assessments as formative assessments to aid in teaching problem solving skills.

Regarding the monitoring growth and facilitating comparisons use (e.g., PISA and NAEP), it would seem that we learn quite a bit with the little p versions of complex problem solving tasks that is useful for educational policy. In PISA, for example, we have learned that although there is a high correlation between problem solving and content test scores (Mathematics, Reading, and Science) nations differentiate themselves on their relative accomplishment in problem solving, in ways that are not predictable from scores on the other assessments. The U.S.’s relatively strong performance on problem solving (compared to its performance in the other content domains), or more generally, the relatively strong performance in problem solving of the high-functioning economies, suggests that students from those economies are acquiring more general problem solving skills (relative to other skills) in school compared to students from weaker economies. That difference does not show up so clearly in content skills. The fact that girls tend to outperform boys in all nations on collaborative problem solving is also an interesting finding. Would we learn more with more complex problem solving tasks, that is, BIG P tasks? Perhaps, but the evidence that would warrant such an investment that it would take to find out is lacking.

Regarding the selection use of problem solving: The high correlation between problem solving and other measures suggests that problem solving assessments, which might have greater face validity, and perhaps content validity, might be viable substitutes for traditional IQ tests. However, regarding the question of whether BIG P assessments should replace little p ones, it is the case that selection applications have historically been sensitive to the time devoted to them. Thus, here again, the evidence that longer, more complex problem solving assessments would add value beyond the shorter versions, particularly given the additional testing time required, is lacking. 

With regard to the formative assessment use, it is here that the more complex, longer-lasting BIG P microworlds are likely to provide unique value beyond what can be had with their shorter, little p counterparts. Formative assessment and student diagnosis uses are ideally suited to longer explorations from students engaging with microworlds. Such applications are currently being researched in various domains [60], although not general complex problem solving, to our knowledge. In any event, this is likely to remain a fruitful pursuit. 

Designing complex problem solving tasks that best meet these various purposes will be challenging, because the purposes are different. Reckase [61] provided a compelling perspective on this design challenge, distinguishing two perspectives on test design.^10^ One he referred to as the psychological perspective, and the other, the educational perspective. The psychological perspective leads to tests that use homogeneous tasks to place people along a continuum. Trait and ability testing fits this notion, and much of test theory assumes this perspective—selection and monitoring uses align best with this perspective. 

In contrast, the educational perspective employs a domain sampling approach to indicate the amount of the domain the test taker has mastered. Simple examples are spelling or vocabulary tests in which words are randomly sampled from a dictionary, but more complex examples can be drawn from any domain, as long as there is a sampling strategy that adequately represents the domain. Reckase [61] argued that monitoring growth aligns with a unidimensional view and the continuum model, but diagnostic uses (determining on which topics students are doing well and poorly), and formative assessment uses (using the test to teach the material) align with a multidimensional view and the domain-sampling approach. Further, Reckase argued that to make good instructional (formative) items requires more complex items than are typically used. The BIG P, complex problem solving microworld approach, as outlined by Dorner and Funke [58] would seem to be well suited to this task.

If complex problem solving can be thought of as a combination of general fluid ability and knowledge, then a hybrid continuum and domain-sampling model might be a useful assessment and reporting strategy. Complex problem solving tasks in various domains could be developed, which would enable reporting on both the general complex problem-solving skill, and the domain-specific aspects of it ([61] provides several examples of how these can be combined).

## 6. Can Complex Problem Solving Skills Be Developed?

There are two persistent myths on the development and education of general cognitive ability. Complex problem solving skill certainly is a kind of general cognitive ability, particularly insofar as it largely reflects general fluid (Gf) ability [29]. One myth is that general cognitive ability is immutable. This was an argument presented by Jensen [62] Hernstein and Murray [63] and others, but it has attained the status of conventional wisdom. The argument for its immutability is typically based on two kinds of findings. One is the strength of test-retest correlations over time, such as the finding that IQ tested at age 11 had a correlation of 0.54 (0.67 when adjusted for range restriction) with IQ tested at age 90 [64]. The other is on the heritability of IQ [65] based on twin studies (identical twins reared apart), which typically estimate the heritability of intelligence to be from 20% in infancy to 80% in later adulthood.

However, both these findings leave plenty of room for environmental effects on general cognitive ability. The Flynn effect, that IQ has increased by about three points (0.2 standard deviations) per decade [66], is one piece of evidence. Another is the effects of school on IQ, which tends to be about 2–5 points per year of school (e.g., [67,68]). This estimate is based on studies that vary widely in their methodology, and on the degree to which the evidence may be considered causal rather than correlational (the strongest causal evidence may be the natural experiment on changing the age of mandatory schooling [69]), but the fact that the estimate is approximately the same regardless of method increases confidence that there is an effect of schooling on IQ. Another piece of evidence is the fact that achievement test scores, which are not assumed to be immutable (they are used in school accountability, for example, [70]), show the same test–retest stability as general cognitive ability tests do [71]. 

The other persistent myth is that even if general cognitive skills are directly trained, that training will not transfer. This idea comes from several sources. One is a body of literature in experimental psychology that illustrates the difficulty of transfer from one setting to another. A classic study [72] showed that reading a story about a military strategy (separate, surround, and converge) did not help students solve a tumor problem that could be solved by an analogous strategy. There are many illustrations of this phenomenon in real life contexts, such as shoppers who are poor at standardized math problems doing well in calculating good deals in the supermarket [73], or bettors who perform complex mental calculations to assess race-track odds performing poorly on standardized tests [74]. This has led to the situated cognition view [75] that knowledge is bound to the context in which it is acquired, making transfer difficult or impossible.

If the situated learning perspective is correct (along with the related concept of authentic assessment [76]), that would bring into question the benefits of using the short problem solving measures used in PISA, and would suggest that the benefits of using even the longer ones advocated by Dorner and Funke [58] were limited. However, Anderson, Reder, and Simon [77] challenged the situated learning perspective, arguing that there were many demonstrations of transfer of arithmetic skills from the classroom to the real world, or transfer of learning from say one text editor to another. They argued that training abstract principles, particularly when combined with concrete examples, was a powerful means of preparing students for future unpredictable performance tasks. 

The view that transfer is impossible is belied by substantial and varied evidence from different corners as well. In the economics literature [78] it can be shown that workers accumulate knowledge and skill (human capital) in an occupation, which is reflected in their growing earnings. When they switch occupations, that acquired knowledge and skill goes with them. The degree of earnings loss experienced is directly related to the similarity of the old and new occupations, a kind of portable skills transfer gradient. In organizations, over $125 billion is spent annually on training, a colossal waste if transfer does not occur. However, meta-analyses have shown that training in organizations does transfer [79] and the substantial literature that exists focusses on the conditions that foster transfer. Those conditions include trainee characteristics such as cognitive ability and conscientiousness, as well as being motivated; supportive work environments; and training interventions that promote knowledge, self-efficacy, and broad skills such as leadership and perhaps, complex problem solving. In education, there have been several reports that assume transfer and have focused on the learning and instructional conditions that facilitate it [12,14]. 

If we accept the notion that complex problem solving skill can be developed, and that it can transfer to real-world problem solving, a question is how best to teach it and how best to monitor its development over time. There was considerable research in the 1980s that explored the value of direct instruction of general problem-solving skills [80,81,82]. Despite some successes [83], much of this line of research fell out of favor for not showing large gains in general problem-solving skills. This led to a movement back towards domain-specific (curricular-focused) instruction. However, with the new-found emphasis on transfer and domain-general abilities, such as problem solving in PISA, or NAEP’s 2014-2016 technology and engineering literacy assessment [84] or student learning outcomes assessment in higher education [85] there may be a renewed interest in direct instruction efforts. Direct problem-solving instruction may be particularly acceptable if nested in a formative instructional context, as suggested in the previous section [61].

## 7. Conclusions

Economic inequality is recognized as a barrier to economic growth and access to quality education [86]. In addition, wealth inequality has been increasing over the past 30 years [87]. Americans tolerate the problem, perhaps because they fail to recognize its magnitude; when given a choice, they dramatically (92% vs. 8%) prefer the wealth distribution of Sweden (36% for the top quintile, 11% for the bottom) over that of the U.S. (84% for the top quintile, 0.1% for the bottom) when those distributions are unlabeled [88]. Various methods have been proposed to address the problem of income and wealth inequality, including progressive income taxation, estate taxation, more open immigration policies, strengthened unions, financial literacy, increased social spending (health and welfare), and pension reform [3]. However, increased education has long been considered the “great equalizer” and, in this commentary, I review some of the evidence on the economic returns to investments in education. There are social, cultural, and health benefits as well. Piketty argued that “in the long run, the best way to reduce inequalities with respect to labor as well as to increase the average productivity of the labor force and the overall growth of the economy is surely to invest in education” [3] (pp. 306–307). Policy makers acknowledge this role of education and therefore continue to support increased educational attainment goals. 

A question is the mechanism by which education delivers these benefits. A widely accepted view is that of a race between education and technology in which technology change fuels economic growth, but also “creates winners and losers” leading to increased inequality [89]. However, “if workers have flexible skills and if the educational infrastructure expands sufficiently, the supply of skills will increase as demand (due to technology changes) increases for them” [89] (p. 26).

What does it mean to have flexible skills? Domain-specific (curricular) skills are important, but, in this commentary I have tried to make a case for the importance of general, domain-independent skills, in particular, complex problem solving. Complex problem-solving skill is a name for a construct used in the workforce literature as a characterization of certain job skills. It is also used in the educational testing and cognitive psychological literatures to characterize the abilities required to solve certain kinds of problems. There are debates in the literature on the boundaries of this definition and the best methods for assessing complex problem solving, but a useful definition involves the ability to solve novel, ill-defined problems in complex, real-world settings. 

Employers seek and reward individuals possessing complex problem-solving skills. Due to technology advances, it is likely that such skills will remain valued, and perhaps increase in value, particularly in combination with communication skills. This suggests that collaborative problem-solving skill is likely to be an important skill for the future workforce. In recognition of this prospect, assessments of collaborative problem-solving skill have been developed already in PISA [49], and such assessments are being planned for NAEP [90] (see [91], for a discussion of associated assessment and measurement issues). 

A wide range of tasks has been put forward for measuring complex problem solving, and it can be argued that the most appropriate task will depend on the particular use—student or employee selection, student development monitoring, or formative assessment. Relatively short, psychometrically efficient tasks are required for the former two uses, but longer, microworld-based tasks [58] may be usefully employed in a formative assessment context. Doing so may entail new task design, analysis, and reporting strategies as outlined by Reckase [61]. It is important for schools at all levels, K-12, community college, career and technical education, and college and university, to recognize the importance of general, complex problem-solving skills for students as part of a strategy to prepare students for the workforce and to thereby reduce the insidious effects of wealth concentration on future opportunities for all.

## Figures and Tables

**Table 1:** Most important skills sought by employers.

NACE 2017 ^a^	“Importance” ^b^	McKinsey ^c^	“Importance” ^d^
Ability to work in a team	78%	Work ethic	80%
Problem-solving skills	77%	Teamwork	79%
Written communication skills	75%	Oral communications	73%
Strong work ethic	72%	Local language	73%
Verbal communication skills	71%	Hands-on-training	69%
Leadership	69%	Problem solving	66%
Initiative	66%	Written communications	64%

Sources: National Association of Colleges and Employers ([23], Figure 2). McKinsey Center for Government [24] (Exhibit 15, p. 44). ^a^ N = 169 US employers, 17% return rate; ^b^ a Survey question is to rate the importance of candidate skills/qualities on a 5-point scale, where 1 = Not at all important, 2 = Not very important, 3 = Somewhat important, 4 = Very important, and 5 = extremely important; ^c^ N = 2832 employers, stratified by sector, size, and distributed across nine countries (Brazil, Germany, India, Mexico, Morocco, Saudi Arabia, Turkey, UK, and USA); ^d^ Survey question is “Please rate how important these skills are for new hires to have in order to be effective at your company…on a scale of 0 to 10, where…10 means extremely important”; Listed is percentage responding 8 or higher out of 10.

## Footnotes

1 Card [7] identified several approaches to estimating causal effects of education, including use of instrumental variables (e.g., minimum school leaving age, tuition costs, and geographic school proximity) and within family (e.g., sibling and twin) comparisons of members with different amounts of education.

2 There is a potential third alternative: years in school or a degree signals to potential employers that a person who achieved this level of education has the general cognitive ability, communication skills, and conscientiousness to have made it this far [10]). Discussion of this issue lies outside the scope of this commentary [11].

3 This is the definition of CPS skills; O*NET [21] also provides the more specific definition of complex problem solving as “identifying complex problems and reviewing related information to develop and evaluate options and implement solutions.”

4 On average, two thirds of the 900 plus occupations are updated each year.

5 Job analysts make independent judgments about the importance and level of skills required for each occupation; there have been 16 ratings cycles which enables calculating interrater reliabilities. For CPS importance, single-rater reliabilities, ICC(C,1) = 0.37, 8-rater reliabilities, ICC(C,8) = 0.83; for CPS level, ICC(C,1) = 0.52, ICC(C,8) = 0.90 [28], Table 3, p. 8. These tend towards the lower end of agreement, with categories such as equipment maintenance, science, troubleshooting, and operation and control at the higher end of agreement.

6 In addition to complex problem solving skills, cross-functional skills also include the categories of social skills, technical skills, systems skills, and resource management skills, each of which includes 3–11 subskills; cross-functional skills are members of a larger category of worker requirements, which also include basic skills, knowledge, and education. Worker requirements are related to worker characteristics, which include abilities, occupational interests, work values, and work styles, each of which has numerous subcategories.

7 OECD [35] also provides a useful projection methodology based on cross-walking job tasks with skills measured in PIAAC, including problem solving, concluding that only a small percentage of workers use skills daily that go beyond current computer capabilities, suggesting a possible mistargeting of education.

8 According to David Leonhardt [36,37], at a 1992 conference on the economy convened by Bill Clinton shortly after his election, no one mentioned the Internet.

9 All of the analytic problems listed here have associated Wikipedia articles (https://en.wikipedia.org/wiki/<problem-name>).

10 These are related to the formative–reflective measurement model distinction in psychometrics, see [29], for a discussion of how this is related to two perspectives on measuring complex problem solving.

## Appendix A. Definitions of Problem Solving in PISA and PIAAC

**PISA 2003**

“Problem solving is an individual’s capacity to use cognitive processes to confront and resolve real, cross-disciplinary situations where the solution path is not immediately obvious and where the literacy domains or curricula areas that might be applicable are not within a single domain of mathematics, science or reading” [47] (p. 154).

**PISA 2012**

“Problem solving competency is an individual’s capacity to engage in cognitive processing to understand and resolve problem situations where a method of solution is not immediately obvious. It includes the willingness to engage with such situations in order to achieve one’s potential as a constructive and reflective citizen” [53] (p. 12).

“For the purposes of the PISA 2012 problem solving assessment, the processes involved in problem solving are taken to be Exploring and understanding; Representing and formulating; Planning and executing; and Monitoring and reflecting” [53] (p. 20–21).

**PISA 2015**

“Three major collaborative problem-solving competencies are identified and defined for measurement in the assessment. These three major CPS competencies are crossed with the four major individual problem-solving processes to form a matrix of specific skills. The specific skills have associated actions, processes and strategies that define what it means for the student to be competent…The three major CPS competencies are… (1) Establishing and maintaining shared understanding… (2) Taking appropriate action to solve the problem… (3) Establishing and maintaining group organization” [48] (pp. 12–13).

**PIAAC**

“In PIAAC, problem solving in technology-rich environments is defined as: using digital technology, communication tools and networks to acquire and evaluate information, communicate with others and perform practical tasks. The first PIAAC problem solving survey will focus on the abilities to solve problems for personal, work and civic purposes by setting up appropriate goals and plans, accessing and making use of information through computers and computer networks” [53] (p. 47).
